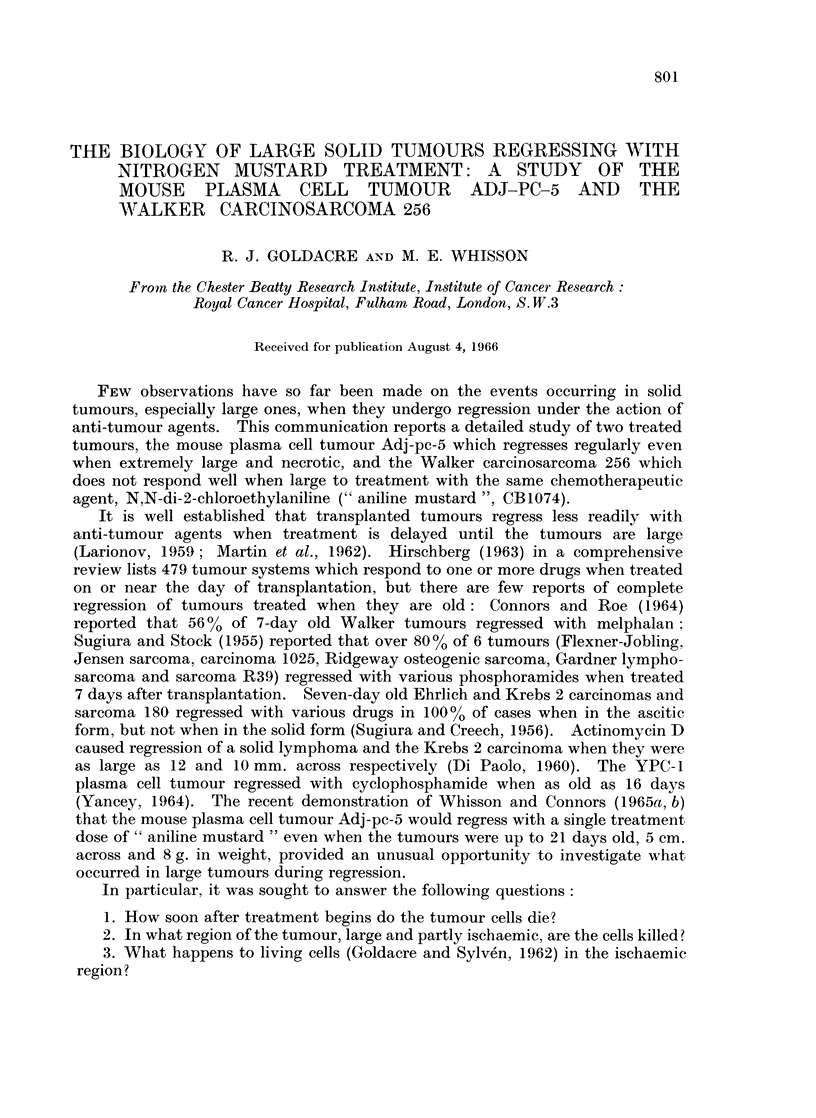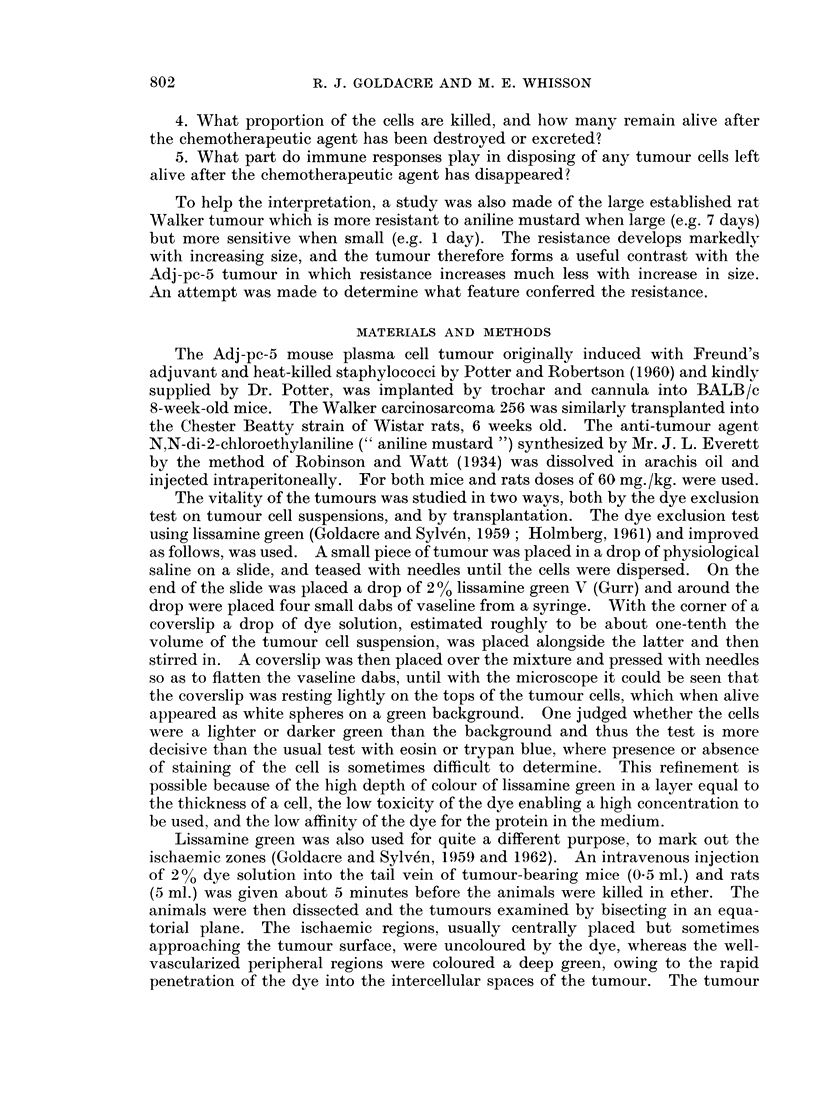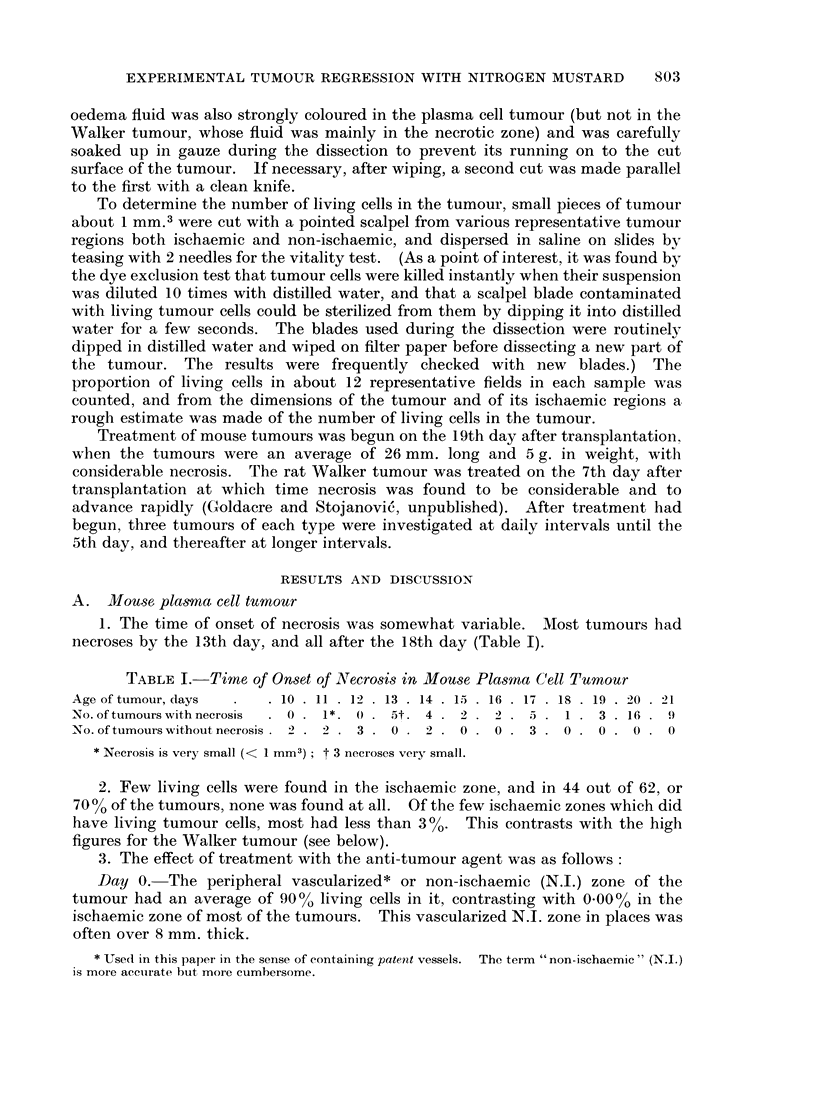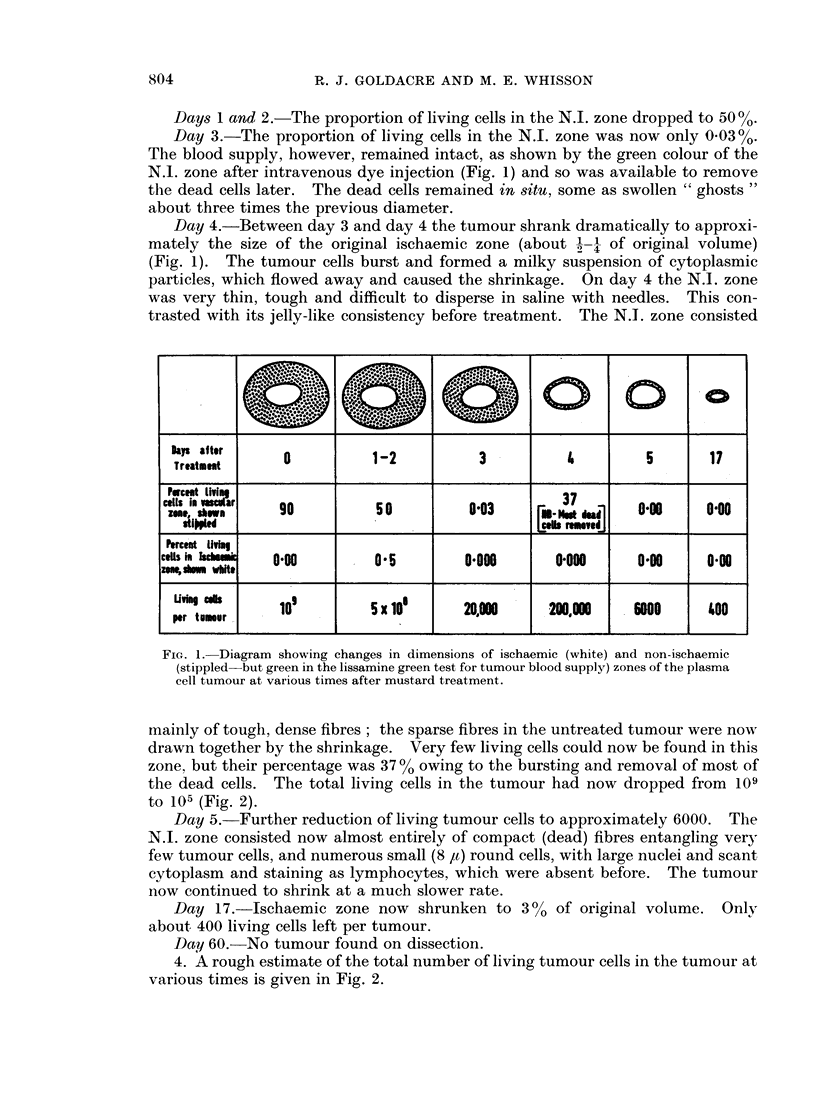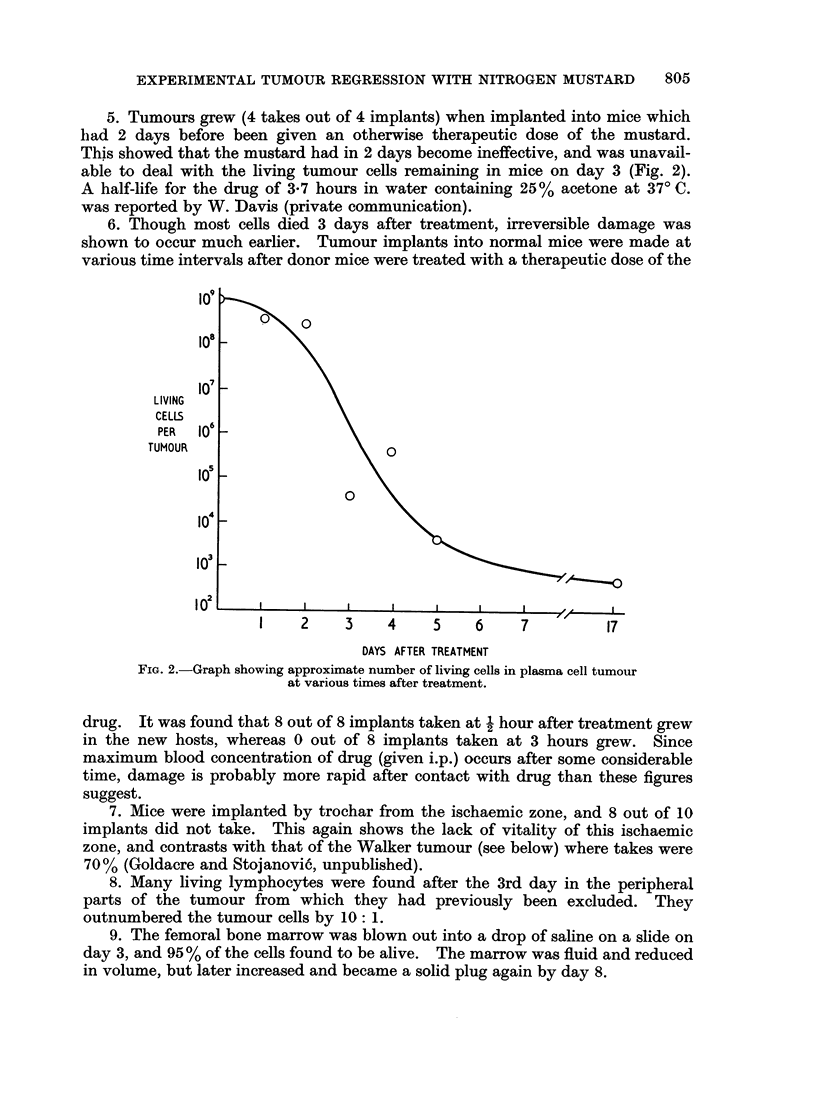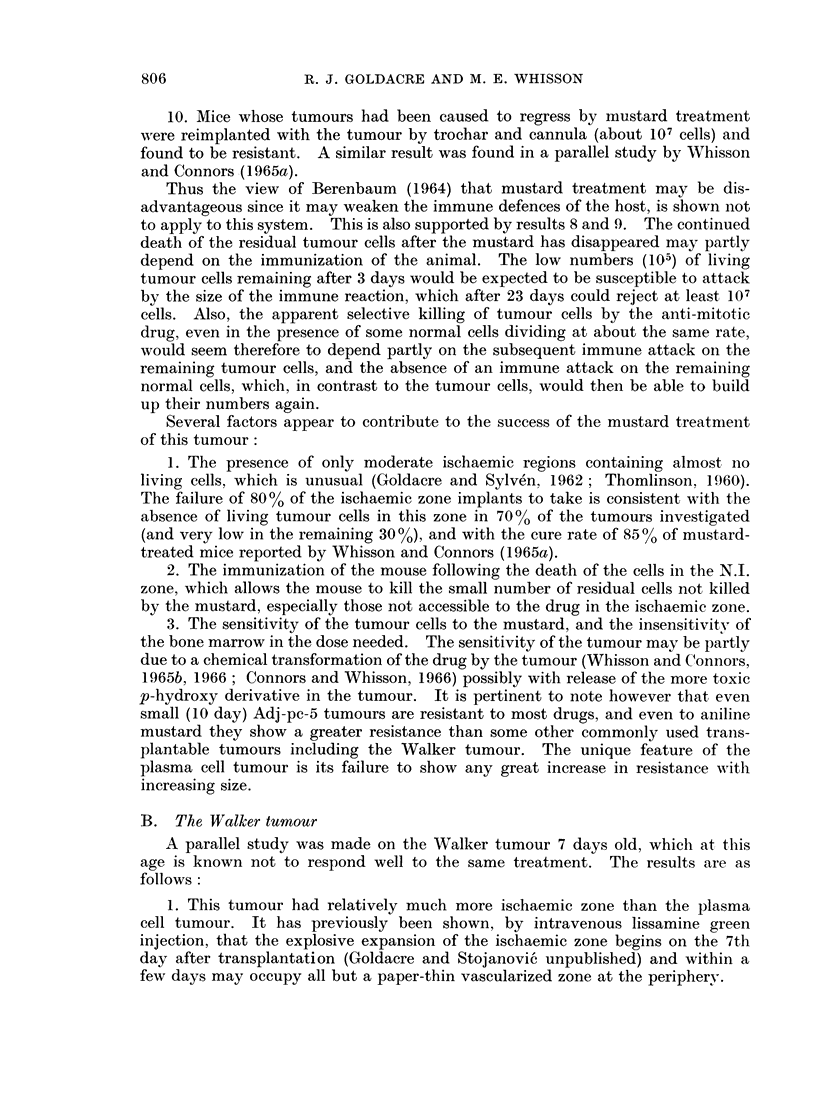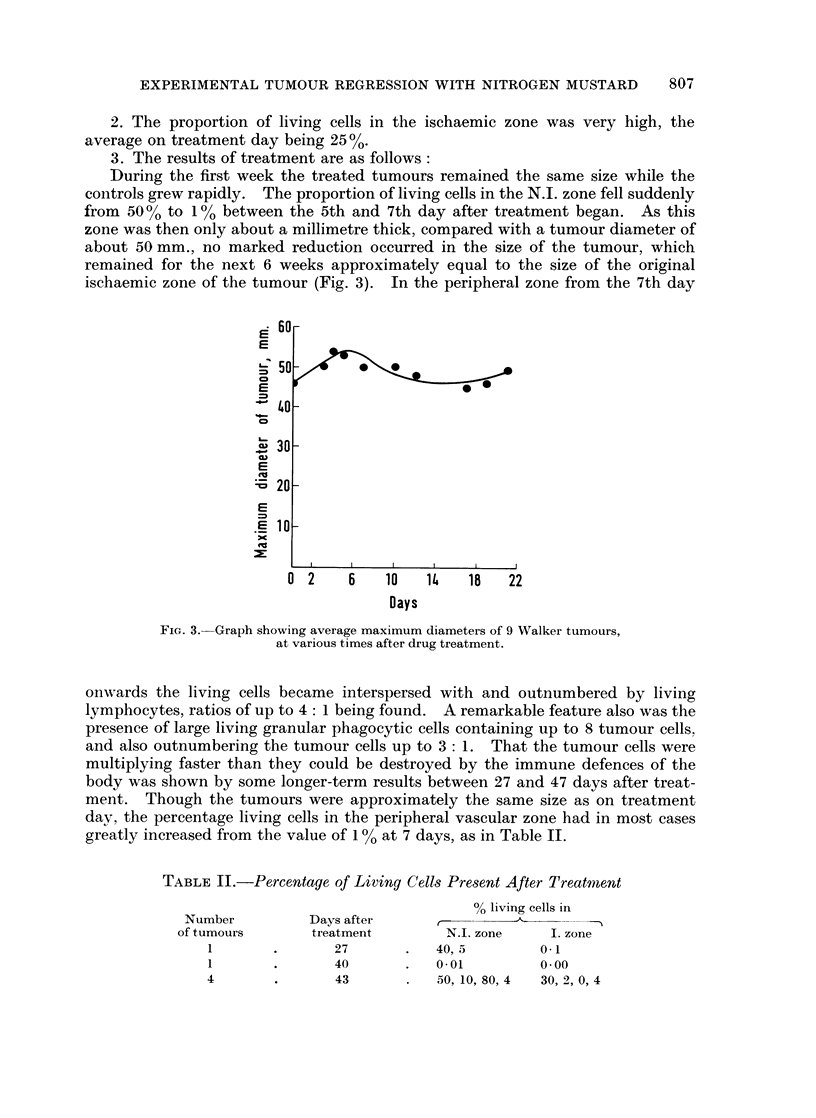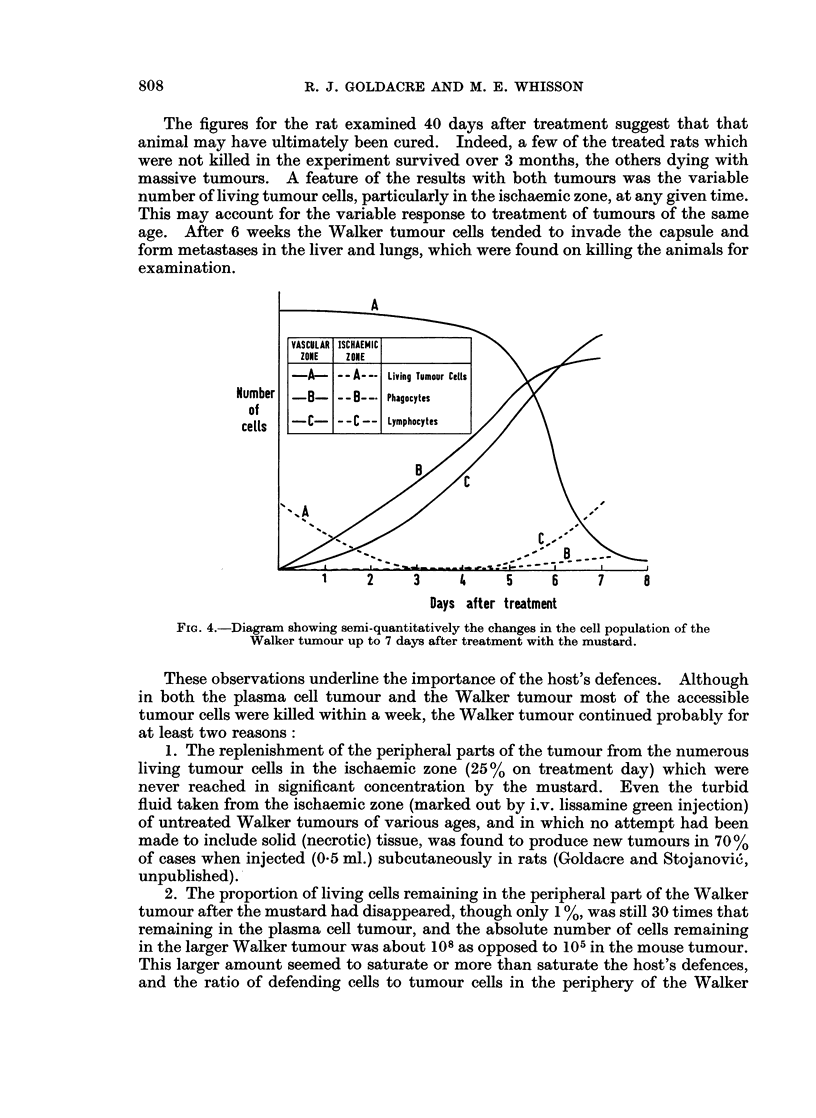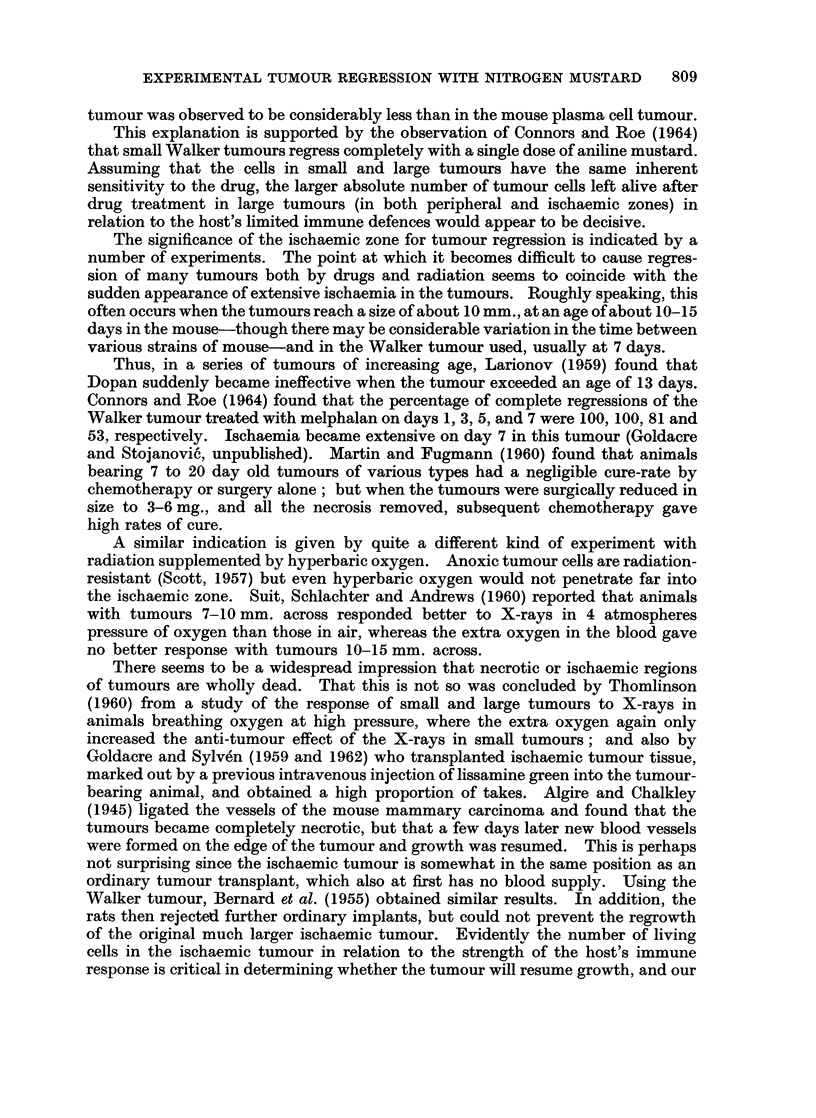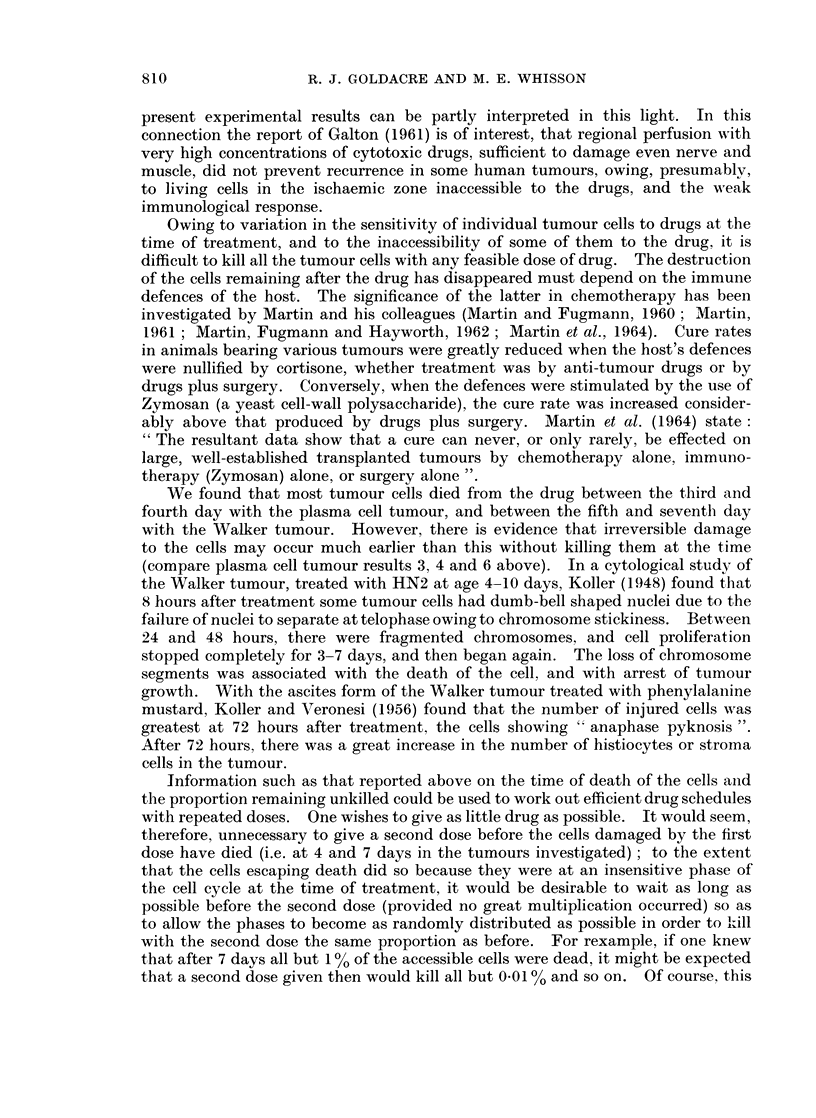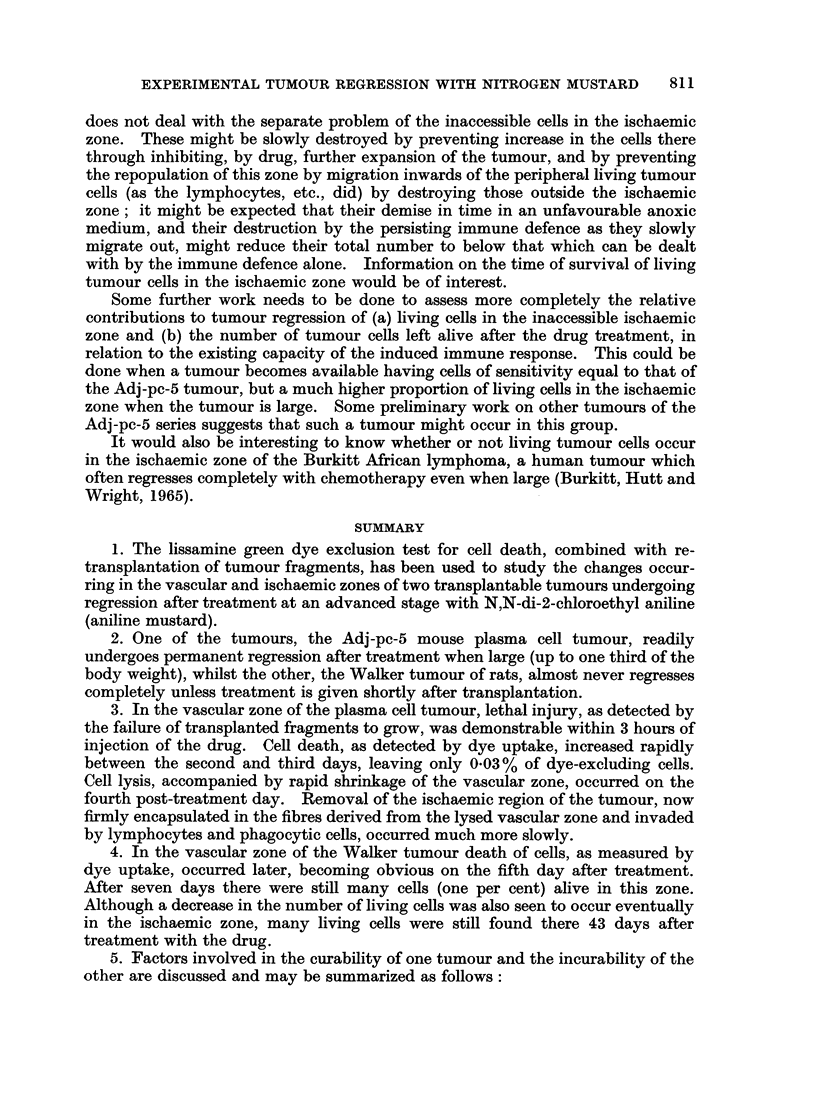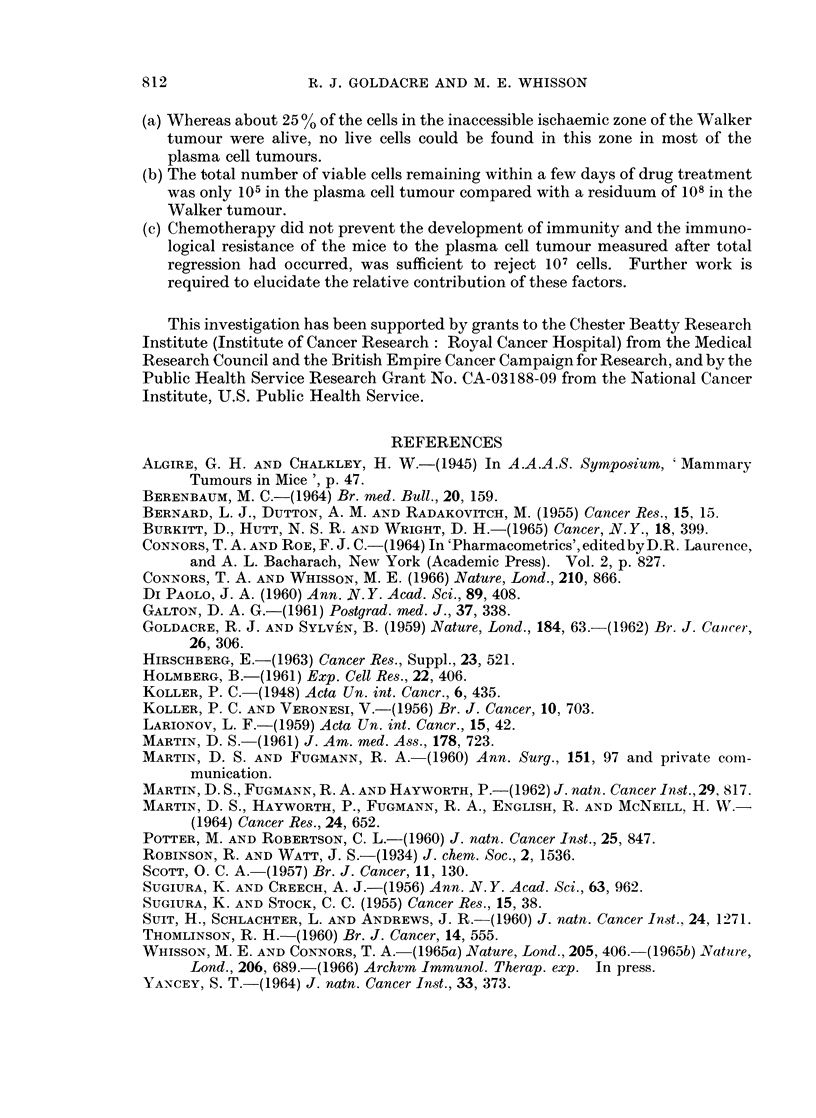# The biology of large solid tumours regressing with nitrogen mustard treatment: a study of the mouse plasma cell tumour Adj-pc-5 and the Walker carcinosarcoma 256.

**DOI:** 10.1038/bjc.1966.91

**Published:** 1966-12

**Authors:** R. J. Goldacre, M. E. Whisson


### THE BIOLOGY OF LARGE SOLID TUMOURS REGRESSING WITH NITROGEN MUSTARD TREATMENT: A STUDY OF THE MOUSE PLASMA CELL TUMOUR ADJ–PC–5 AND THE WALKER CARCINOSARCOMA 256

R. J. GOLDACRE AND M. E. WHISSON

*From the Chester Beatty Research Institute, Institute of Cancer Research : Royal Cancer Hospital, Fulham Road, London, S.W.3*

Received for publication August 4, 1966

FEW observations have so far been made on the events occurring in solid tumours, especially large ones, when they undergo regression under the action of anti-tumour agents. This communication reports a detailed study of two treated tumours, the mouse plasma cell tumour Adj-pc-5 which regresses regularly even when extremely large and necrotic, and the Walker carcinosarcoma 256 which does not respond well when large to treatment with the same chemotherapeutic agent, N,N-di-2-chloroethylaniline (" aniline mustard ", CB1074).

It is well established that transplanted tumours regress less readily with anti-tumour agents when treatment is delayed until the tumours are large (Larionov, 1959 ; Martin *et al.*, 1962). Hirschberg (1963) in a comprehensive review lists 479 tumour systems which respond to one or more drugs when treated on or near the day of transplantation, but there are few reports of complete regression of tumours treated when they are old : Connors and Roe (1964) reported that 56% of 7-day old Walker tumours regressed with melphalan ; Sugiura and Stock (1955) reported that over 80% of 6 tumours (Flexner-Jobling, Jensen sarcoma, carcinoma 1025, Ridgeway osteogenic sarcoma, Gardner lymphosarcoma and sarcoma R39) regressed with various phosphoramides when treated 7 days after transplantation. Seven-day old Ehrlich and Krebs 2 carcinomas and sarcoma 180 regressed with various drugs in 100% of cases when in the ascitic form, but not when in the solid form (Sugiura and Creech, 1956). Actinomycin D caused regression of a solid lymphoma and the Krebs 2 carcinoma when they were as large as 12 and 10 mm. across respectively (Di Paolo, 1960). The YPC-1 plasma cell tumour regressed with cyclophosphamide when as old as 16 days (Yancey, 1964). The recent demonstration of Whisson and Connors (1965*a*, *b*) that the mouse plasma cell tumour Adj-pc-5 would regress with a single treatment dose of " aniline mustard " even when the tumours were up to 21 days old, 5 cm. across and 8 g. in weight, provided an unusual opportunity to investigate what occurred in large tumours during regression.

In particular, it was sought to answer the following questions :

1. How soon after treatment begins do the tumour cells die?
2. In what region of the tumour, large and partly ischaemic, are the cells killed?
3. What happens to living cells (Goldacre and Sylvén, 1962) in the ischaemic region?

4. What proportion of the cells are killed, and how many remain alive after the chemotherapeutic agent has been destroyed or excreted?

5. What part do immune responses play in disposing of any tumour cells left alive after the chemotherapeutic agent has disappeared?

To help the interpretation, a study was also made of the large established rat Walker tumour which is more resistant to aniline mustard when large (e.g. 7 days) but more sensitive when small (e.g. 1 day). The resistance develops markedly with increasing size, and the tumour therefore forms a useful contrast with the Adj-pc-5 tumour in which resistance increases much less with increase in size. An attempt was made to determine what feature conferred the resistance.

### MATERIALS AND METHODS

The Adj-pc-5 mouse plasma cell tumour originally induced with Freund's adjuvant and heat-killed staphylococci by Potter and Robertson (1960) and kindly supplied by Dr. Potter, was implanted by trochar and cannula into BALB/c 8-week-old mice. The Walker carcinosarcoma 256 was similarly transplanted into the Chester Beatty strain of Wistar rats, 6 weeks old. The anti-tumour agent N,N-di-2-chloroethylaniline (" aniline mustard ") synthesized by Mr. J. L. Everett by the method of Robinson and Watt (1934) was dissolved in arachis oil and injected intraperitoneally. For both mice and rats doses of 60 mg./kg. were used.

The vitality of the tumours was studied in two ways, both by the dye exclusion test on tumour cell suspensions, and by transplantation. The dye exclusion test using lissamine green (Goldacre and Sylvén, 1959 ; Holmberg, 1961) and improved as follows, was used. A small piece of tumour was placed in a drop of physiological saline on a slide, and teased with needles until the cells were dispersed. On the end of the slide was placed a drop of 2% lissamine green V (Gurr) and around the drop were placed four small dabs of vaseline from a syringe. With the corner of a coverslip a drop of dye solution, estimated roughly to be about one-tenth the volume of the tumour cell suspension, was placed alongside the latter and then stirred in. A coverslip was then placed over the mixture and pressed with needles so as to flatten the vaseline dabs, until with the microscope it could be seen that the coverslip was resting lightly on the tops of the tumour cells, which when alive appeared as white spheres on a green background. One judged whether the cells were a lighter or darker green than the background and thus the test is more decisive than the usual test with eosin or trypan blue, where presence or absence of staining of the cell is sometimes difficult to determine. This refinement is possible because of the high depth of colour of lissamine green in a layer equal to the thickness of a cell, the low toxicity of the dye enabling a high concentration to be used, and the low affinity of the dye for the protein in the medium.

Lissamine green was also used for quite a different purpose, to mark out the ischaemic zones (Goldacre and Sylvén, 1959 and 1962). An intravenous injection of 2% dye solution into the tail vein of tumour-bearing mice (0·5 ml.) and rats (5 ml.) was given about 5 minutes before the animals were killed in ether. The animals were then dissected and the tumours examined by bisecting in an equatorial plane. The ischaemic regions, usually centrally placed but sometimes approaching the tumour surface, were uncoloured by the dye, whereas the well-vascularized peripheral regions were coloured a deep green, owing to the rapid penetration of the dye into the intercellular spaces of the tumour. The tumour oedema fluid was also strongly coloured in the plasma cell tumour (but not in the Walker tumour, whose fluid was mainly in the necrotic zone) and was carefully soaked up in gauze during the dissection to prevent its running on to the cut surface of the tumour. If necessary, after wiping, a second cut was made parallel to the first with a clean knife.

To determine the number of living cells in the tumour, small pieces of tumour about 1 mm.$^3$ were cut with a pointed scalpel from various representative tumour regions both ischaemic and non-ischaemic, and dispersed in saline on slides by teasing with 2 needles for the vitality test. (As a point of interest, it was found by the dye exclusion test that tumour cells were killed instantly when their suspension was diluted 10 times with distilled water, and that a scalpel blade contaminated with living tumour cells could be sterilized from them by dipping it into distilled water for a few seconds. The blades used during the dissection were routinely dipped in distilled water and wiped on filter paper before dissecting a new part of the tumour. The results were frequently checked with new blades.) The proportion of living cells in about 12 representative fields in each sample was counted, and from the dimensions of the tumour and of its ischaemic regions a rough estimate was made of the number of living cells in the tumour.

Treatment of mouse tumours was begun on the 19th day after transplantation, when the tumours were an average of 26 mm. long and 5 g. in weight, with considerable necrosis. The rat Walker tumour was treated on the 7th day after transplantation at which time necrosis was found to be considerable and to advance rapidly (Goldacre and Stojanović, unpublished). After treatment had begun, three tumours of each type were investigated at daily intervals until the 5th day, and thereafter at longer intervals.

### RESULTS AND DISCUSSION

### A. *Mouse plasma cell tumour*

1. The time of onset of necrosis was somewhat variable. Most tumours had necroses by the 13th day, and all after the 18th day (Table I).

TABLE I.—*Time of Onset of Necrosis in Mouse Plasma Cell Tumour*

| | | | | | | | | | | | | |
|---|---|---|---|---|---|---|---|---|---|---|---|---|
| Age of tumour, days | 10 | 11 | 12 | 13 | 14 | 15 | 16 | 17 | 18 | 19 | 20 | 21 |
| No. of tumours with necrosis | 0 | 1* | 0 | 5† | 4 | 2 | 2 | 5 | 1 | 3 | 16 | 9 |
| No. of tumours without necrosis | 2 | 2 | 3 | 0 | 2 | 0 | 0 | 3 | 0 | 0 | 0 | 0 |

* Necrosis is very small (< 1 mm$^3$) ; † 3 necroses very small.

2. Few living cells were found in the ischaemic zone, and in 44 out of 62, or 70% of the tumours, none was found at all. Of the few ischaemic zones which did have living tumour cells, most had less than 3%. This contrasts with the high figures for the Walker tumour (see below).

3. The effect of treatment with the anti-tumour agent was as follows :

*Day* 0.—The peripheral vascularized* or non-ischaemic (N.I.) zone of the tumour had an average of 90% living cells in it, contrasting with 0·00% in the ischaemic zone of most of the tumours. This vascularized N.I. zone in places was often over 8 mm. thick.

* Used in this paper in the sense of containing *patent* vessels. The term "non-ischaemic" (N.I.) is more accurate but more cumbersome.

*Days* 1 *and* 2.—The proportion of living cells in the N.I. zone dropped to 50%.

*Day* 3.—The proportion of living cells in the N.I. zone was now only 0·03%. The blood supply, however, remained intact, as shown by the green colour of the N.I. zone after intravenous dye injection (Fig. 1) and so was available to remove the dead cells later. The dead cells remained *in situ*, some as swollen " ghosts " about three times the previous diameter.

*Day* 4.—Between day 3 and day 4 the tumour shrank dramatically to approximately the size of the original ischaemic zone (about $\frac{1}{2}$–$\frac{1}{4}$ of original volume) (Fig. 1). The tumour cells burst and formed a milky suspension of cytoplasmic particles, which flowed away and caused the shrinkage. On day 4 the N.I. zone was very thin, tough and difficult to disperse in saline with needles. This contrasted with its jelly-like consistency before treatment. The N.I. zone consisted

| Days after Treatment | 0 | 1-2 | 3 | 4 | 5 | 17 |
|---|---|---|---|---|---|---|
| Percent living cells in vascular zone, shown stippled | 90 | 50 | 0·03 | 37 [NB-Most dead cells removed] | 0·00 | 0·00 |
| Percent living cells in Ischaemic zone, shown white | 0·00 | 0·5 | 0·000 | 0·000 | 0·00 | 0·00 |
| Living cells per tumour | $10^9$ | $5 \times 10^8$ | 20,000 | 200,000 | 6000 | 400 |

FIG. 1.—Diagram showing changes in dimensions of ischaemic (white) and non-ischaemic (stippled—but green in the lissamine green test for tumour blood supply) zones of the plasma cell tumour at various times after mustard treatment.

mainly of tough, dense fibres ; the sparse fibres in the untreated tumour were now drawn together by the shrinkage. Very few living cells could now be found in this zone, but their percentage was 37% owing to the bursting and removal of most of the dead cells. The total living cells in the tumour had now dropped from $10^9$ to $10^5$ (Fig. 2).

*Day* 5.—Further reduction of living tumour cells to approximately 6000. The N.I. zone consisted now almost entirely of compact (dead) fibres entangling very few tumour cells, and numerous small (8 $\mu$) round cells, with large nuclei and scant cytoplasm and staining as lymphocytes, which were absent before. The tumour now continued to shrink at a much slower rate.

*Day* 17.—Ischaemic zone now shrunken to 3% of original volume. Only about 400 living cells left per tumour.

*Day* 60.—No tumour found on dissection.

4. A rough estimate of the total number of living tumour cells in the tumour at various times is given in Fig. 2.

5. Tumours grew (4 takes out of 4 implants) when implanted into mice which had 2 days before been given an otherwise therapeutic dose of the mustard. This showed that the mustard had in 2 days become ineffective, and was unavailable to deal with the living tumour cells remaining in mice on day 3 (Fig. 2). A half-life for the drug of 3·7 hours in water containing 25% acetone at 37° C. was reported by W. Davis (private communication).

6. Though most cells died 3 days after treatment, irreversible damage was shown to occur much earlier. Tumour implants into normal mice were made at various time intervals after donor mice were treated with a therapeutic dose of the

FIG. 2.—Graph showing approximate number of living cells in plasma cell tumour at various times after treatment.

drug. It was found that 8 out of 8 implants taken at ½ hour after treatment grew in the new hosts, whereas 0 out of 8 implants taken at 3 hours grew. Since maximum blood concentration of drug (given i.p.) occurs after some considerable time, damage is probably more rapid after contact with drug than these figures suggest.

7. Mice were implanted by trochar from the ischaemic zone, and 8 out of 10 implants did not take. This again shows the lack of vitality of this ischaemic zone, and contrasts with that of the Walker tumour (see below) where takes were 70% (Goldacre and Stojanović, unpublished).

8. Many living lymphocytes were found after the 3rd day in the peripheral parts of the tumour from which they had previously been excluded. They outnumbered the tumour cells by 10 : 1.

9. The femoral bone marrow was blown out into a drop of saline on a slide on day 3, and 95% of the cells found to be alive. The marrow was fluid and reduced in volume, but later increased and became a solid plug again by day 8.

10. Mice whose tumours had been caused to regress by mustard treatment were reimplanted with the tumour by trochar and cannula (about $10^7$ cells) and found to be resistant. A similar result was found in a parallel study by Whisson and Connors (1965*a*).

Thus the view of Berenbaum (1964) that mustard treatment may be disadvantageous since it may weaken the immune defences of the host, is shown not to apply to this system. This is also supported by results 8 and 9. The continued death of the residual tumour cells after the mustard has disappeared may partly depend on the immunization of the animal. The low numbers ($10^5$) of living tumour cells remaining after 3 days would be expected to be susceptible to attack by the size of the immune reaction, which after 23 days could reject at least $10^7$ cells. Also, the apparent selective killing of tumour cells by the anti-mitotic drug, even in the presence of some normal cells dividing at about the same rate, would seem therefore to depend partly on the subsequent immune attack on the remaining tumour cells, and the absence of an immune attack on the remaining normal cells, which, in contrast to the tumour cells, would then be able to build up their numbers again.

Several factors appear to contribute to the success of the mustard treatment of this tumour :

1. The presence of only moderate ischaemic regions containing almost no living cells, which is unusual (Goldacre and Sylvén, 1962 ; Thomlinson, 1960). The failure of 80% of the ischaemic zone implants to take is consistent with the absence of living tumour cells in this zone in 70% of the tumours investigated (and very low in the remaining 30%), and with the cure rate of 85% of mustard-treated mice reported by Whisson and Connors (1965*a*).

2. The immunization of the mouse following the death of the cells in the N.I. zone, which allows the mouse to kill the small number of residual cells not killed by the mustard, especially those not accessible to the drug in the ischaemic zone.

3. The sensitivity of the tumour cells to the mustard, and the insensitivity of the bone marrow in the dose needed. The sensitivity of the tumour may be partly due to a chemical transformation of the drug by the tumour (Whisson and Connors, 1965*b*, 1966 ; Connors and Whisson, 1966) possibly with release of the more toxic *p*-hydroxy derivative in the tumour. It is pertinent to note however that even small (10 day) Adj-pc-5 tumours are resistant to most drugs, and even to aniline mustard they show a greater resistance than some other commonly used transplantable tumours including the Walker tumour. The unique feature of the plasma cell tumour is its failure to show any great increase in resistance with increasing size.

B. *The Walker tumour*

A parallel study was made on the Walker tumour 7 days old, which at this age is known not to respond well to the same treatment. The results are as follows :

1. This tumour had relatively much more ischaemic zone than the plasma cell tumour. It has previously been shown, by intravenous lissamine green injection, that the explosive expansion of the ischaemic zone begins on the 7th day after transplantation (Goldacre and Stojanović unpublished) and within a few days may occupy all but a paper-thin vascularized zone at the periphery.

2. The proportion of living cells in the ischaemic zone was very high, the average on treatment day being 25%.

3. The results of treatment are as follows :

During the first week the treated tumours remained the same size while the controls grew rapidly. The proportion of living cells in the N.I. zone fell suddenly from 50% to 1% between the 5th and 7th day after treatment began. As this zone was then only about a millimetre thick, compared with a tumour diameter of about 50 mm., no marked reduction occurred in the size of the tumour, which remained for the next 6 weeks approximately equal to the size of the original ischaemic zone of the tumour (Fig. 3). In the peripheral zone from the 7th day

FIG. 3.—Graph showing average maximum diameters of 9 Walker tumours, at various times after drug treatment.

onwards the living cells became interspersed with and outnumbered by living lymphocytes, ratios of up to 4 : 1 being found. A remarkable feature also was the presence of large living granular phagocytic cells containing up to 8 tumour cells, and also outnumbering the tumour cells up to 3 : 1. That the tumour cells were multiplying faster than they could be destroyed by the immune defences of the body was shown by some longer-term results between 27 and 47 days after treatment. Though the tumours were approximately the same size as on treatment day, the percentage living cells in the peripheral vascular zone had in most cases greatly increased from the value of 1% at 7 days, as in Table II.

TABLE II.—*Percentage of Living Cells Present After Treatment*

| Number of tumours | Days after treatment | % living cells in N.I. zone | % living cells in I. zone |
|---|---|---|---|
| 1 | 27 | 40, 5 | 0·1 |
| 1 | 40 | 0·01 | 0·00 |
| 4 | 43 | 50, 10, 80, 4 | 30, 2, 0, 4 |

The figures for the rat examined 40 days after treatment suggest that that animal may have ultimately been cured. Indeed, a few of the treated rats which were not killed in the experiment survived over 3 months, the others dying with massive tumours. A feature of the results with both tumours was the variable number of living tumour cells, particularly in the ischaemic zone, at any given time. This may account for the variable response to treatment of tumours of the same age. After 6 weeks the Walker tumour cells tended to invade the capsule and form metastases in the liver and lungs, which were found on killing the animals for examination.

FIG. 4.—Diagram showing semi-quantitatively the changes in the cell population of the Walker tumour up to 7 days after treatment with the mustard.

These observations underline the importance of the host's defences. Although in both the plasma cell tumour and the Walker tumour most of the accessible tumour cells were killed within a week, the Walker tumour continued probably for at least two reasons :

1. The replenishment of the peripheral parts of the tumour from the numerous living tumour cells in the ischaemic zone (25% on treatment day) which were never reached in significant concentration by the mustard. Even the turbid fluid taken from the ischaemic zone (marked out by i.v. lissamine green injection) of untreated Walker tumours of various ages, and in which no attempt had been made to include solid (necrotic) tissue, was found to produce new tumours in 70% of cases when injected (0·5 ml.) subcutaneously in rats (Goldacre and Stojanović, unpublished).

2. The proportion of living cells remaining in the peripheral part of the Walker tumour after the mustard had disappeared, though only 1%, was still 30 times that remaining in the plasma cell tumour, and the absolute number of cells remaining in the larger Walker tumour was about $10^8$ as opposed to $10^5$ in the mouse tumour. This larger amount seemed to saturate or more than saturate the host's defences, and the ratio of defending cells to tumour cells in the periphery of the Walker tumour was observed to be considerably less than in the mouse plasma cell tumour.

This explanation is supported by the observation of Connors and Roe (1964) that small Walker tumours regress completely with a single dose of aniline mustard. Assuming that the cells in small and large tumours have the same inherent sensitivity to the drug, the larger absolute number of tumour cells left alive after drug treatment in large tumours (in both peripheral and ischaemic zones) in relation to the host's limited immune defences would appear to be decisive.

The significance of the ischaemic zone for tumour regression is indicated by a number of experiments. The point at which it becomes difficult to cause regression of many tumours both by drugs and radiation seems to coincide with the sudden appearance of extensive ischaemia in the tumours. Roughly speaking, this often occurs when the tumours reach a size of about 10 mm., at an age of about 10–15 days in the mouse—though there may be considerable variation in the time between various strains of mouse—and in the Walker tumour used, usually at 7 days.

Thus, in a series of tumours of increasing age, Larionov (1959) found that Dopan suddenly became ineffective when the tumour exceeded an age of 13 days. Connors and Roe (1964) found that the percentage of complete regressions of the Walker tumour treated with melphalan on days 1, 3, 5, and 7 were 100, 100, 81 and 53, respectively. Ischaemia became extensive on day 7 in this tumour (Goldacre and Stojanović, unpublished). Martin and Fugmann (1960) found that animals bearing 7 to 20 day old tumours of various types had a negligible cure-rate by chemotherapy or surgery alone ; but when the tumours were surgically reduced in size to 3–6 mg., and all the necrosis removed, subsequent chemotherapy gave high rates of cure.

A similar indication is given by quite a different kind of experiment with radiation supplemented by hyperbaric oxygen. Anoxic tumour cells are radiation-resistant (Scott, 1957) but even hyperbaric oxygen would not penetrate far into the ischaemic zone. Suit, Schlachter and Andrews (1960) reported that animals with tumours 7–10 mm. across responded better to X-rays in 4 atmospheres pressure of oxygen than those in air, whereas the extra oxygen in the blood gave no better response with tumours 10–15 mm. across.

There seems to be a widespread impression that necrotic or ischaemic regions of tumours are wholly dead. That this is not so was concluded by Thomlinson (1960) from a study of the response of small and large tumours to X-rays in animals breathing oxygen at high pressure, where the extra oxygen again only increased the anti-tumour effect of the X-rays in small tumours ; and also by Goldacre and Sylvén (1959 and 1962) who transplanted ischaemic tumour tissue, marked out by a previous intravenous injection of lissamine green into the tumour-bearing animal, and obtained a high proportion of takes. Algire and Chalkley (1945) ligated the vessels of the mouse mammary carcinoma and found that the tumours became completely necrotic, but that a few days later new blood vessels were formed on the edge of the tumour and growth was resumed. This is perhaps not surprising since the ischaemic tumour is somewhat in the same position as an ordinary tumour transplant, which also at first has no blood supply. Using the Walker tumour, Bernard *et al.* (1955) obtained similar results. In addition, the rats then rejected further ordinary implants, but could not prevent the regrowth of the original much larger ischaemic tumour. Evidently the number of living cells in the ischaemic tumour in relation to the strength of the host's immune response is critical in determining whether the tumour will resume growth, and our present experimental results can be partly interpreted in this light. In this connection the report of Galton (1961) is of interest, that regional perfusion with very high concentrations of cytotoxic drugs, sufficient to damage even nerve and muscle, did not prevent recurrence in some human tumours, owing, presumably, to living cells in the ischaemic zone inaccessible to the drugs, and the weak immunological response.

Owing to variation in the sensitivity of individual tumour cells to drugs at the time of treatment, and to the inaccessibility of some of them to the drug, it is difficult to kill all the tumour cells with any feasible dose of drug. The destruction of the cells remaining after the drug has disappeared must depend on the immune defences of the host. The significance of the latter in chemotherapy has been investigated by Martin and his colleagues (Martin and Fugmann, 1960 ; Martin, 1961 ; Martin, Fugmann and Hayworth, 1962 ; Martin *et al.*, 1964). Cure rates in animals bearing various tumours were greatly reduced when the host's defences were nullified by cortisone, whether treatment was by anti-tumour drugs or by drugs plus surgery. Conversely, when the defences were stimulated by the use of Zymosan (a yeast cell-wall polysaccharide), the cure rate was increased considerably above that produced by drugs plus surgery. Martin *et al.* (1964) state : " The resultant data show that a cure can never, or only rarely, be effected on large, well-established transplanted tumours by chemotherapy alone, immunotherapy (Zymosan) alone, or surgery alone ".

We found that most tumour cells died from the drug between the third and fourth day with the plasma cell tumour, and between the fifth and seventh day with the Walker tumour. However, there is evidence that irreversible damage to the cells may occur much earlier than this without killing them at the time (compare plasma cell tumour results 3, 4 and 6 above). In a cytological study of the Walker tumour, treated with HN2 at age 4–10 days, Koller (1948) found that 8 hours after treatment some tumour cells had dumb-bell shaped nuclei due to the failure of nuclei to separate at telophase owing to chromosome stickiness. Between 24 and 48 hours, there were fragmented chromosomes, and cell proliferation stopped completely for 3–7 days, and then began again. The loss of chromosome segments was associated with the death of the cell, and with arrest of tumour growth. With the ascites form of the Walker tumour treated with phenylalanine mustard, Koller and Veronesi (1956) found that the number of injured cells was greatest at 72 hours after treatment, the cells showing " anaphase pyknosis ". After 72 hours, there was a great increase in the number of histiocytes or stroma cells in the tumour.

Information such as that reported above on the time of death of the cells and the proportion remaining unkilled could be used to work out efficient drug schedules with repeated doses. One wishes to give as little drug as possible. It would seem, therefore, unnecessary to give a second dose before the cells damaged by the first dose have died (i.e. at 4 and 7 days in the tumours investigated) ; to the extent that the cells escaping death did so because they were at an insensitive phase of the cell cycle at the time of treatment, it would be desirable to wait as long as possible before the second dose (provided no great multiplication occurred) so as to allow the phases to become as randomly distributed as possible in order to kill with the second dose the same proportion as before. For rexample, if one knew that after 7 days all but 1% of the accessible cells were dead, it might be expected that a second dose given then would kill all but 0·01% and so on. Of course, this does not deal with the separate problem of the inaccessible cells in the ischaemic zone. These might be slowly destroyed by preventing increase in the cells there through inhibiting, by drug, further expansion of the tumour, and by preventing the repopulation of this zone by migration inwards of the peripheral living tumour cells (as the lymphocytes, etc., did) by destroying those outside the ischaemic zone ; it might be expected that their demise in time in an unfavourable anoxic medium, and their destruction by the persisting immune defence as they slowly migrate out, might reduce their total number to below that which can be dealt with by the immune defence alone. Information on the time of survival of living tumour cells in the ischaemic zone would be of interest.

Some further work needs to be done to assess more completely the relative contributions to tumour regression of (a) living cells in the inaccessible ischaemic zone and (b) the number of tumour cells left alive after the drug treatment, in relation to the existing capacity of the induced immune response. This could be done when a tumour becomes available having cells of sensitivity equal to that of the Adj-pc-5 tumour, but a much higher proportion of living cells in the ischaemic zone when the tumour is large. Some preliminary work on other tumours of the Adj-pc-5 series suggests that such a tumour might occur in this group.

It would also be interesting to know whether or not living tumour cells occur in the ischaemic zone of the Burkitt African lymphoma, a human tumour which often regresses completely with chemotherapy even when large (Burkitt, Hutt and Wright, 1965).

### SUMMARY

1. The lissamine green dye exclusion test for cell death, combined with retransplantation of tumour fragments, has been used to study the changes occurring in the vascular and ischaemic zones of two transplantable tumours undergoing regression after treatment at an advanced stage with N,N-di-2-chloroethyl aniline (aniline mustard).

2. One of the tumours, the Adj-pc-5 mouse plasma cell tumour, readily undergoes permanent regression after treatment when large (up to one third of the body weight), whilst the other, the Walker tumour of rats, almost never regresses completely unless treatment is given shortly after transplantation.

3. In the vascular zone of the plasma cell tumour, lethal injury, as detected by the failure of transplanted fragments to grow, was demonstrable within 3 hours of injection of the drug. Cell death, as detected by dye uptake, increased rapidly between the second and third days, leaving only 0·03% of dye-excluding cells. Cell lysis, accompanied by rapid shrinkage of the vascular zone, occurred on the fourth post-treatment day. Removal of the ischaemic region of the tumour, now firmly encapsulated in the fibres derived from the lysed vascular zone and invaded by lymphocytes and phagocytic cells, occurred much more slowly.

4. In the vascular zone of the Walker tumour death of cells, as measured by dye uptake, occurred later, becoming obvious on the fifth day after treatment. After seven days there were still many cells (one per cent) alive in this zone. Although a decrease in the number of living cells was also seen to occur eventually in the ischaemic zone, many living cells were still found there 43 days after treatment with the drug.

5. Factors involved in the curability of one tumour and the incurability of the other are discussed and may be summarized as follows :

(a) Whereas about 25% of the cells in the inaccessible ischaemic zone of the Walker tumour were alive, no live cells could be found in this zone in most of the plasma cell tumours.
(b) The total number of viable cells remaining within a few days of drug treatment was only $10^5$ in the plasma cell tumour compared with a residuum of $10^8$ in the Walker tumour.
(c) Chemotherapy did not prevent the development of immunity and the immunological resistance of the mice to the plasma cell tumour measured after total regression had occurred, was sufficient to reject $10^7$ cells. Further work is required to elucidate the relative contribution of these factors.

This investigation has been supported by grants to the Chester Beatty Research Institute (Institute of Cancer Research : Royal Cancer Hospital) from the Medical Research Council and the British Empire Cancer Campaign for Research, and by the Public Health Service Research Grant No. CA-03188-09 from the National Cancer Institute, U.S. Public Health Service.

### REFERENCES

ALGIRE, G. H. AND CHALKLEY, H. W.—(1945) In *A.A.A.S. Symposium*, ' Mammary Tumours in Mice ', p. 47.
BERENBAUM, M. C.—(1964) *Br. med. Bull.*, **20**, 159.
BERNARD, L. J., DUTTON, A. M. AND RADAKOVITCH, M. (1955) *Cancer Res.*, **15**, 15.
BURKITT, D., HUTT, N. S. R. AND WRIGHT, D. H.—(1965) *Cancer, N.Y.*, **18**, 399.
CONNORS, T. A. AND ROE, F. J. C.—(1964) In 'Pharmacometrics', edited by D.R. Laurence, and A. L. Bacharach, New York (Academic Press). Vol. 2, p. 827.
CONNORS, T. A. AND WHISSON, M. E. (1966) *Nature, Lond.*, **210**, 866.
DI PAOLO, J. A. (1960) *Ann. N.Y. Acad. Sci.*, **89**, 408.
GALTON, D. A. G.—(1961) *Postgrad. med. J.*, **37**, 338.
GOLDACRE, R. J. AND SYLVÉN, B. (1959) *Nature, Lond.*, **184**, 63.—(1962) *Br. J. Cancer*, **26**, 306.
HIRSCHBERG, E.—(1963) *Cancer Res.*, Suppl., **23**, 521.
HOLMBERG, B.—(1961) *Exp. Cell Res.*, **22**, 406.
KOLLER, P. C.—(1948) *Acta Un. int. Cancr.*, **6**, 435.
KOLLER, P. C. AND VERONESI, V.—(1956) *Br. J. Cancer*, **10**, 703.
LARIONOV, L. F.—(1959) *Acta Un. int. Cancr.*, **15**, 42.
MARTIN, D. S.—(1961) *J. Am. med. Ass.*, **178**, 723.
MARTIN, D. S. AND FUGMANN, R. A.—(1960) *Ann. Surg.*, **151**, 97 and private communication.
MARTIN, D. S., FUGMANN, R. A. AND HAYWORTH, P.—(1962) *J. natn. Cancer Inst.*, **29**, 817.
MARTIN, D. S., HAYWORTH, P., FUGMANN, R. A., ENGLISH, R. AND MCNEILL, H. W.—(1964) *Cancer Res.*, **24**, 652.
POTTER, M. AND ROBERTSON, C. L.—(1960) *J. natn. Cancer Inst.*, **25**, 847.
ROBINSON, R. AND WATT, J. S.—(1934) *J. chem. Soc.*, **2**, 1536.
SCOTT, O. C. A.—(1957) *Br. J. Cancer*, **11**, 130.
SUGIURA, K. AND CREECH, A. J.—(1956) *Ann. N.Y. Acad. Sci.*, **63**, 962.
SUGIURA, K. AND STOCK, C. C. (1955) *Cancer Res.*, **15**, 38.
SUIT, H., SCHLACHTER, L. AND ANDREWS, J. R.—(1960) *J. natn. Cancer Inst.*, **24**, 1271.
THOMLINSON, R. H.—(1960) *Br. J. Cancer*, **14**, 555.
WHISSON, M. E. AND CONNORS, T. A.—(1965*a*) *Nature, Lond.*, **205**, 406.—(1965*b*) *Nature, Lond.*, **206**, 689.—(1966) *Archvm Immunol. Therap. exp.* In press.
YANCEY, S. T.—(1964) *J. natn. Cancer Inst.*, **33**, 373.